# Cohort study of intervened functionally univentricular heart in England and Wales (2000–2018)

**DOI:** 10.1136/heartjnl-2021-319677

**Published:** 2021-10-27

**Authors:** Elena Hadjicosta, Rodney Franklin, Anna Seale, Oliver Stumper, Victor Tsang, David R Anderson, Christina Pagel, Sonya Crowe, Ferran Espuny Pujol, Deborah Ridout, Kate L Brown

**Affiliations:** 1 Clinical Operational Research Unit, Department of Mathematics, University College London, London, UK; 2 Paediatric Cardiology, Royal Brompton and Harefield NHS Trust, London, UK; 3 Paediatric Cardiology, Birmingham Children’s Hospital, Birmingham, UK; 4 Heart and Lung Division, Great Ormond Street Hospital, London, UK; 5 Institute of Cardiovascular Science, University College London, London, UK; 6 Paediatric Cardiac Surgery, Evelina London Children's Healthcare, London, UK; 7 University College London Institute of Child Health, London, UK; 8 NIHR Great Ormond Street Hospital Biomedical Research Centre, London, UK

**Keywords:** heart defects, congenital, outcome assessment, health care

## Abstract

**Objective:**

Given the paucity of long-term outcome data for complex congenital heart disease (CHD), we aimed to describe the treatment pathways and survival for patients who started interventions for functionally univentricular heart (FUH) conditions, excluding hypoplastic left heart syndrome.

**Methods:**

We performed a retrospective cohort study using all procedure records from the National Congenital Heart Diseases Audit for children born in 2000–2018. The primary outcome was mortality, ascertained from the Office for National Statistics in 2020.

**Results:**

Of 53 615 patients, 1557 had FUH: 55.9% were boys and 67.4% were of White ethnic groups. The largest diagnostic categories were tricuspid atresia (28.9%), double inlet left ventricle (21.0%) and unbalanced atrioventricular septal defect (AVSD) (15.2%). The ages at staged surgery were: initial palliation 11.5 (IQR 5.5–43.5) days, cavopulmonary shunt 9.2 (IQR 6.0–17.1) months and Fontan 56.2 (IQR 45.5–70.3) months. The median follow-up time was 10.8 (IQR 7.0–14.9) years and the 1, 5 and 10-year survival rates after initial palliation were 83.6% (95% CI 81.7% to 85.4%), 79.4% (95% CI 77.3% to 81.4%) and 77.2% (95% CI 75.0% to 79.2%), respectively. Higher hazards were present for unbalanced AVSD HR 2.75 (95% CI 1.82 to 4.17), atrial isomerism HR 1.75 (95% CI 1.14 to 2.70) and low weight HR 1.65 (95% CI 1.13 to 2.41), critical illness HR 2.30 (95% CI 1.67 to 3.18) or acquired comorbidities HR 2.71 (95% CI 1.82 to 4.04) at initial palliation.

**Conclusion:**

Although treatment pathways for FUH are complex and variable, nearly 8 out of 10 children survived to 10 years. Longer-term analyses of outcome based on diagnosis (rather than procedure) can inform parents, patients and clinicians, driving practice improvements for complex CHD.

## Introduction

Early operative mortality for paediatric cardiac surgery is very low^1–3^; nonetheless, complexity has increased within the operated population of children with congenital heart disease (CHD).^4^ Late mortalities occur in complex CHD, for which a series of operations and lifelong multidisciplinary care are required.^5–8^ Population-based studies of longer term CHD outcomes are scarce: a systematic review of long-term survival in CHD identified only 16 population-based studies worldwide,^9^ most of which dated before many currently available treatments existed, and very few contained analyses of individual complex CHDs. The most complex group of CHDs are those with only one functional ventricle: importantly, this is a growing population of patients.^10^

Recognising a knowledge gap, we previously used procedure-based records from the UK National Congenital Heart Diseases Audit (NCHDA) linked with patient survival data from the Office for National Statistics (ONS), to describe treatment pathways and long-term outcomes for babies born with a diagnosis of classic hypoplastic left heart syndrome (HLHS).^11 12^ In the current study, given that we already covered HLHS outcomes in our previous paper, we aimed to undertake a cohort study including other types of functionally univentricular heart (FUH). We base our overarching definition of FUH on the consensus description of the International Society for the Nomenclature of Paediatric and Congenital Heart Disease:

The term ‘functionally univentricular heart’ describes a spectrum of congenital cardiac malformations in which the ventricular mass may not readily lend itself to partitioning that commits one ventricular pump to the systemic circulation and another to the pulmonary circulation.^13^

This study focuses on hearts with double inlet atrioventricular connection (both double inlet left ventricle (DILV) and double inlet right ventricle (DIRV)); hearts with absence of one atrioventricular connection (mitral atresia (MA) and tricuspid atresia (TA)); hearts with a common atrioventricular valve and only one completely well-developed ventricle (unbalanced common atrioventricular septal defect (AVSD)); hearts with only one fully well-developed ventricle and atrial isomerism (heterotaxia syndrome, referred to as atrial isomerism in the UK); and, finally, other rare forms of FUH that do not fit in one of these specified major categories.^13^

## Methods

### Study design

This was a retrospective cohort study.

### Data sources

We used all records of cardiac surgical procedures and interventional catheters performed in England and Wales between 1 April 2000 and 31 March 2018, recorded in NCHDA. Data submission to NCHDA is mandatory and subject to external data validation. Each procedure record in NCHDA contains several diagnostic and procedure codes derived from the International Paediatric and Congenital Cardiac Code.^14^ The procedure-based records were linked using pseudonymised patient identifiers (National Health Service (NHS) number, patient hospital identifier, patient name and postcode). In cases of inconsistent data, linkage between procedure records was checked manually.

Patient vital status (dead or alive) was provided at the point of hospital discharge by NCHDA, as obtained from treating centres. Patients’ longer term vital status, including age at death from their death certificates, was obtained from the ONS. We received the ages of surviving patients in November 2020. Any patients who had missing life status with the ONS were treated as lost to follow-up and censored at their most recent discharge age.

### Inclusion and exclusion criteria

As the NCHDA is a procedure-based registry, patients who did not undergo any cardiac procedures do not appear in the data set.

#### Disease level

We identified and excluded patients with classic HLHS,^15^ which we previously described in detail.^11^ We identified and included patients with unequivocal FUH.^15^ We identified and excluded patients with CHD that might be suitable for either biventricular or FUH treatment based on individual patient morphology, for example, pulmonary atresia with intact ventricular septum, which go beyond the scope of this study.

#### Patient level

We excluded patients born before 1 April 2000 to create a data set where the complete procedural history was present. We excluded patients from overseas (including Ireland), Scotland and Northern Ireland because life status was unavailable. We excluded a very small number of patients with clinically significant missing data such that a reliable patient history could not be ascertained.

#### Record level

Only therapeutic cardiovascular surgical procedures (bypass and non-bypass), interventional cardiology and major electrophysiology procedures were included.

### Outcomes

The primary outcome was survival: the status of the patient recorded as dead if designated dead by the treating centre or by ONS life status.

The secondary outcome was occurrence of additional cardiovascular interventions over and above the surgical stages of planned FUH palliation, either by catheter or surgery.

### Data management

We developed a hierarchical algorithm (online supplemental appendix 1) to identify patients with FUH using diagnostic and procedure codes. Then we used a hierarchy to subdivide FUH into atrial isomerism, DILV, DIRV, TA, MA, unbalanced AVSD and ‘other FUH types’ including double outlet right ventricle (DORV).

10.1136/heartjnl-2021-319677.supp1 Supplementary data

### Procedure categorisation

We classified the procedures present in the data set into one of the following groups, based on their sequence in patient management and clinical interpretation (online supplemental appendix 2).

#### Prepathway procedures

Interventions that occurred after the child’s birth (fetal procedures were not included) and before initial palliation (eg, balloon atrial septostomy).

#### Procedures on the established Fontan pathway of palliation for FUH

##### Initial palliation (stage 1)

Procedures that are routinely the first surgical palliation procedure for FUH, including Norwood or Damus surgery, HLHS-type hybrid procedures, coarctation/interrupted arch repairs, procedures to secure pulmonary blood flow (eg, systemic-to-pulmonary arterial shunts) and procedures to protect the pulmonary vascular bed (eg, pulmonary trunk or arterial bands). Given that patients with FUH sometimes have more than one of these procedures, we designated the first instance as the initial palliation, and then subsequent instances as ‘additional cardiac procedure(s)’.^13 16^

##### Cavopulmonary shunt stage (stage 2)

Construction of a bidirectional superior cavopulmonary (Glenn) anastomosis (BCPA), including when contemporaneous with other procedures that are sometimes required (eg, pulmonary arterial reconstruction).^13 16^

##### Comprehensive stage 2

A combination of aortopulmonary amalgamation and augmentation with construction of a BCPA.^13 16^

##### Fontan stage (stage 3)

Total cavopulmonary connection procedures (or Fontan) including when contemporaneous with other procedures that are sometimes required (eg, atrioventricular valve repair).^13 16^

#### Additional cardiac procedures (secondary outcome)

These procedures included surgeries and interventional catheterisations undertaken to augment the baseline staged palliative surgical treatment pathway of patients with FUH for a range of reasons.

### Risk factors

#### Demographics

Gender, ethnicity (NCHDA categories of White, Black, Asian, Mixed, Other or Unknown; we refer to Asian ethnicity as ‘South Asian’ since this represents mainly Pakistani, Bangladeshi or Indian background^17^) and deprivation (assigned using quintiles of the Index of Multiple Deprivation by financial year of procedure^18^).

#### Clinical variables

Antenatal diagnosis, congenital extracardiac comorbidities (eg, congenital lung anomalies) and prematurity (birth at gestation <37 weeks).^19 20^ In addition, the following risk factors were derived at the index procedure: acquired comorbidities (eg, necrotising enterocolitis), increased severity of illness (a need for preoperative ventilation or presence of preoperative shock)^20^ and weight-for-age z-score.^21^ Weight-for-age z-scores outside the range of ±5 were considered anomalous and treated as missing.

### Statistical methods

We created an infographic to show the number of patients on different trajectories in terms of their prepathway and pathway procedures and survival status.

We calculated the median and IQR of age at operation, length of stay, weight and weight-for-age z-score at each operative stage. CIs for in-hospital mortality at each stage of treatment were calculated using the Wilson (score) method.

We explored whether there was an association between occurrence of prepathway procedures and clinical risk factors using Fisher’s exact test.

Survival analysis was initially conducted using the Kaplan-Meier approach, with the primary outcome of death representing failure. We carried out univariable and multivariable Cox regression with the risk factors, which were selected a priori. The risk period for each patient was taken from the time of their index procedure until death or last follow-up. Patients lost to follow-up were treated as censored and those with missing risk factor data were excluded (complete case analysis). The proportional hazards assumption was checked for each factor in turn using statistical tests based on the Schoenfeld residuals. Also, assumption was checked graphically using log-log plots and observed versus predicted survival curves.

For the secondary outcome, we developed three negative binomial regression models for the outcomes of ‘any additional cardiovascular procedures’, ‘additional surgical procedures’ and ‘additional catheter procedures’ and included patient exposure time as an offset. We included an interaction term for increased severity of illness at initial palliation.

We carried out sensitivity analyses to compare the results of the models for the primary and secondary outcomes when all patients with missing data for ethnicity, antenatal diagnosis or deprivation were assigned as having or not the risk factor under consideration.

All statistical analyses were performed with Stata V.15 software (StataCorp, Texas, USA).

### Patient and public involvement

As we have done for HLHS, we will work with the patient and parent organisation ‘Little Hearts Matter’ to share long-term outcomes for children with FUH in their family information.

## Results

### Patient cohort

**Figure 1:**
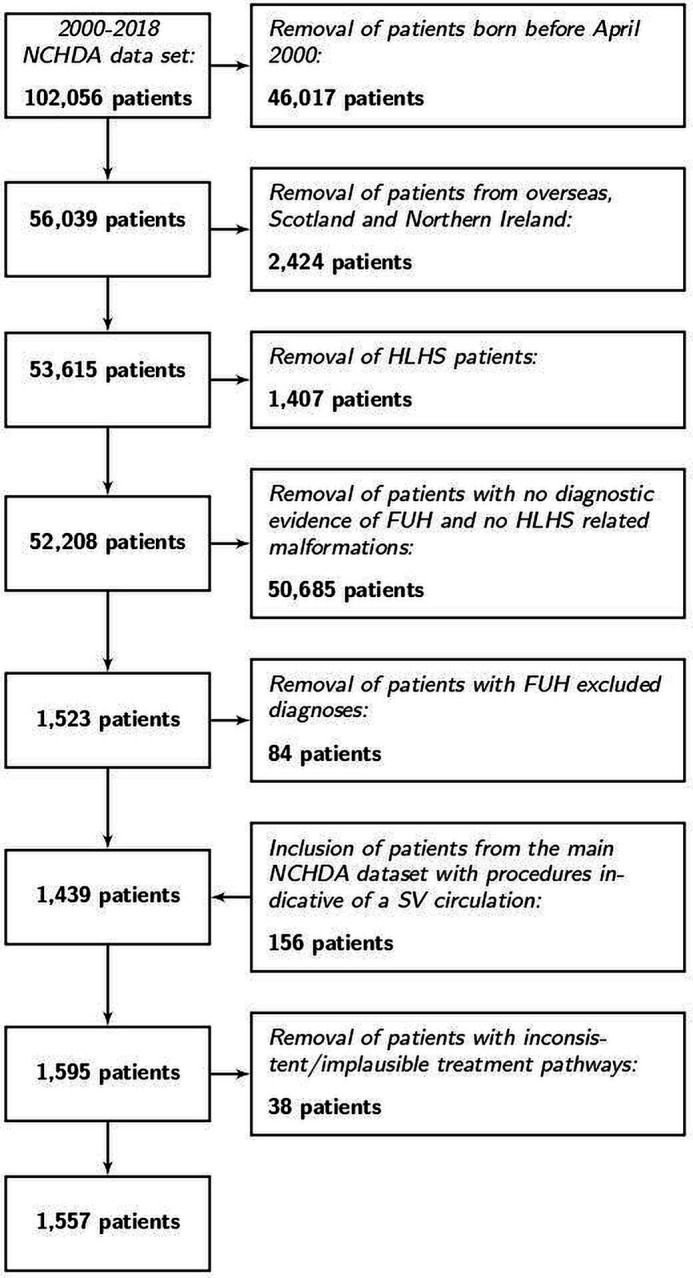
The process of case ascertainment of the study cohort of patients with functionally univentricular heart (FUH) from the National Congenital Heart Diseases Audit (NCHDA) data set with specific exclusions stated at each step. HLHS, hypoplastic left heart syndrome; SV, single ventricle.

Figure 1 shows the number of patients excluded at each stage leading to the final cohort of 1557 patients with FUH. ONS life status was missing for 20 (1.3%) patients in whom we used instead of vital status at discharge from the last procedure-related admission. The few missing data are stated in each table.

### Patient descriptors and risk factors

The risk factors for death at any time point are summarised in table 1, including the breakdown by FUH subtype (14.0% atrial isomerism, 21.0% DILV, 1.0% DIRV, 28.9% TA, 6.7% MA, 15.2% unbalanced AVSD and 13.2% other FUH types including DORV). There were more boys than girls (55.9% and 44.1%, respectively), the most prevalent black and minority ethnic group was South Asian (18.3% of patients) and 34.2% of patients lived at an address represented in the most deprived quintile. The majority of patients with FUH (78.6%) had an antenatal diagnosis, 17.3% had a congenital non-cardiac comorbidity and (table 2) at all surgical stages the patients tended to have low weight for age.

**Table 1:** Frequencies and percentages of risk factors for patients with FUH displayed with the results of the survival models

Risk factor	Frequency	Percentage (%)	Risk factor categorisation in Cox model	Univariable HR (95% CI)	Multivariable HR (95% CI)
**Clinical subgroups**			**Clinical subgroups (Ref: other FUH)**		
FUH with atrial isomerism	218	14.0	FUH with atrial isomerism	1.61 (1.09 to 2.38)	1.75 (1.14 to 2.70)*
Double inlet left ventricle (DILV)	327	21.0	DILV or DIRV	0.52 (0.33 to 0.81)	0.49 (0.30 to 0.83)**
Double inlet right ventricle (DIRV)	16	1.0			
Tricuspid atresia (TA)	450	28.9	Tricuspid atresia	1.19 (0.80 to 1.78)	1.22 (0.78 to 1.92)
			Tricuspid atresia (interaction) follow-up time	0.80 (0.68 to 0.94)	0.79 (0.66 to 0.94)**
Mitral atresia (MA)	104	6.7	Mitral atresia	1.28 (0.78 to 2.10)	1.44 (0.84 to 2.49)
Unbalanced AVSD	236	15.2	Unbalanced AVSD	2.74 (1.91 to 3.94)	2.75 (1.82 to 4.17)***
Other FUH	206	13.2			
**Gender**			**Gender (Ref: female)**		
Male	870	55.9	Male	0.91 (0.74 to 1.12)	0.94 (0.74 to 1.19)
Female	687	44.1			
**Index procedure weight below 2.5 kg**			**Index procedure weight <2.5 kg (Ref: >2.5 kg)**		
No	1357	87.2			
Yes	113	7.3	Yes	1.83 (1.31 to 2.54)	1.65 (1.13 to 2.41)*
Missing	87	5.6			
**Ethnicity**			**Ethnicity (Ref: non-white)**		
White	1050	67.4	White	0.81 (0.65 to 1.02)	0.88 (0.68 to 1.14)
Black	92	5.9			
Asian	285	18.3			
Mixed	7	0.5			
Other	72	4.6			
Missing	51	3.3			
**Antenatal diagnosis**			**Antenatal diagnosis (Ref: none)**		
No	293	18.8			
Yes	1224	78.6	Yes	1.22 (0.92 to 1.62)	1.03 (0.76 to 1.41)
Missing	40	2.6			
**Congenital non-cardiac comorbidity**			**Congenital non-cardiac comorbidity (Ref: none)**		
No	1288	82.7			
Yes	269	17.3	Yes	1.36 (1.05 to 1.75)	0.98 (0.73 to 1.31)
**Prematurity**			**Prematurity (Ref: full term)**		
No	1447	92.9			
Yes	110	7.1	Yes	1.32 (0.91 to 1.92)	0.75 (0.46 to 1.24)
**Index procedure acquired comorbidity**			**Acquired comorbidity (Ref: none)**		
No	1486	95.4			
Yes	71	4.6	Yes	2.88 (2.02 to 4.10)	2.71 (1.82 to 4.04)***
**Index procedure increased severity of illness**			**Increased severity of illness (Ref: none)**		
No	1417	91.0			
Yes	140	9.0	Yes	2.32 (1.74 to 3.11)	2.31 (1.67 to 3.18)***
**Index of Multiple Deprivation (IMD)**			**IMD (Ref: most deprived)**		
Most deprived	533	34.2			
Second most deprived	345	22.2	Second most deprived	0.93 (0.71 to 1.22)	0.88 (0.65 to 1.19)
Mid-deprived	240	15.4	Mid-deprived	0.77 (0.57 to 1.09)	0.74 (0.51 to 1.07)
Second least deprived	171	11.0	Second least deprived	0.63 (0.42 to 0.94)	0.82 (0.52 to 1.27)
Least deprived	156	10.0	Least deprived	0.71 (0.48 to 1.06)	0.85 (0.55 to 1.30)
Missing	112	7.2			

*P<0.05; **p<0.01; ***p<0.001.

AVSD, atrioventricular septal defect; FUH, functionally univentricular heart.

**Table 2:** Frequencies, timing and weights at each stage of the treatment pathway for FUH

Procedure category	Frequency (%)	Median age (IQR)	Median length of stay (IQR)	Median weight (kg) (IQR)	Median weight-for-age z-score (IQR)
Prepathway	212 (13.6)	8.5 (2.5–34.1) days	7.5 (2.5–20.5) days	3.3 (2.8–3.8)	−1.4 (−2.4 to −0.6)
Initial palliation	1263 (81.1)	11.5 (5.5–43.5) days	12.3 (6.5–23.5) days	3.3 (2.9–3.8)	−1.6 (−2.6 to −0.8)
Cavopulmonary shunt stage	1100 (70.6)	9.2 (6.0–17.1) months	6.5 (4.6–10.5) days	7.7 (6.3–9.4)	−1.6 (−2.5 to −0.8)
Fontan stage	814 (52.3)	56.2 (45.5–70.3) months	11.5 (8.5–17.5) days	16.2 (14.4–19.2)	−0.8 (−1.6 to 0.1)
Comprehensive stage 2	102 (6.6)	10.0 (5.8–13.8) months	7.5 (5.5–16.5) days	7.5 (6.0–9.1)	−2.0 (−2.9 to −0.8)
Heart transplant	15 (1.0)	7.7 (1.5–9.7) years	20.5 (15.3–49.5) days	19.8 (8.1–27.0)	−1.8 (−1.9 to −0.5)

There were 11 missing length of stay values (1 for prepathway; 6 for stage 1; 3 for stage 2; 1 for stage 3).

There were 128 missing weights/weight-for-age z-scores (9 for prepathway; 74 for stage 1; 39 for stage 2; 4 for Fontan; 2 for comprehensive stage 2).

FUH, functionally univentricular heart.

### Description of treatment pathways

**Figure 2:**
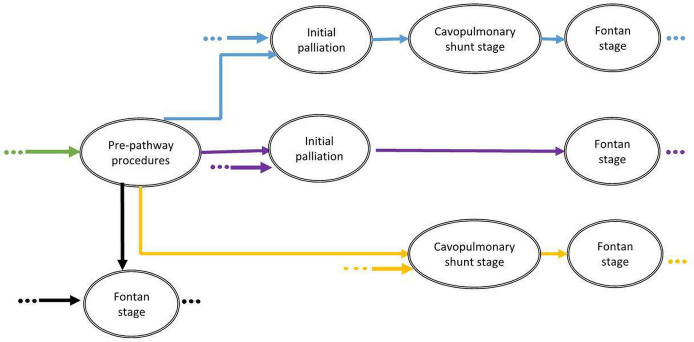
The possible treatment pathways for patients with functionally univentricular heart, excluding hypoplastic left heart syndrome, in England and Wales.

**Figure 3:**
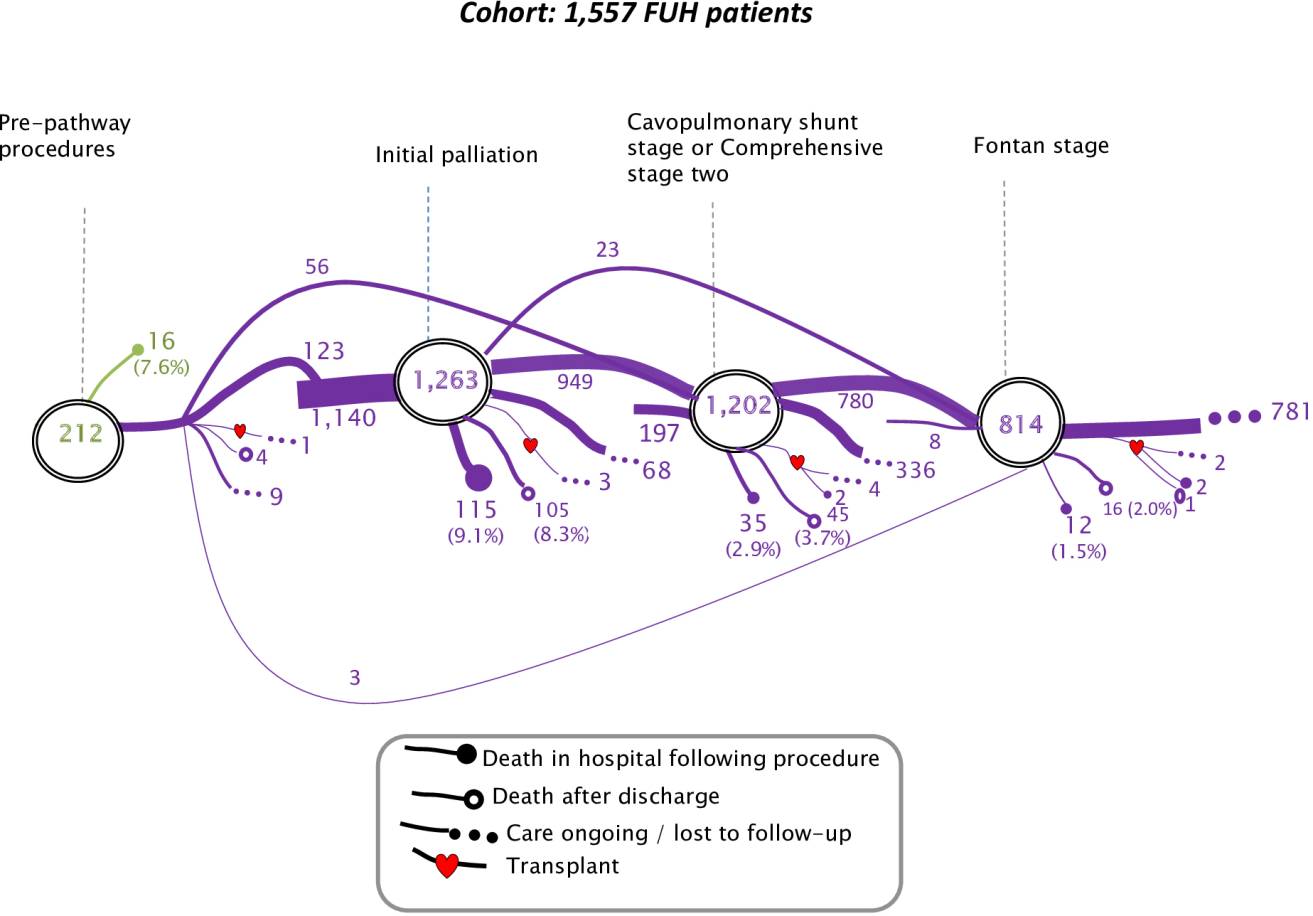
Treatment pathways and outcomes for patients born with functionally univentricular heart (FUH), excluding hypoplastic left heart syndrome, between 2000 and 2018, who underwent any interventions for their disease in England and Wales.

Figure 2 summarises the possible treatment pathways experienced by patients with FUH. Figure 3 depicts the treatment pathways and captures the number of patients who had undergone each surgical stage at the time the data set was analysed. Of the 1557 patients with FUH overall, 212 (13.6%) had a prepathway intervention, 1263 (81.1%) had an initial palliation, 1202 (77.2%) had a cavopulmonary shunt stage (including 102 comprehensive stage 2) and 814 (52.3%) had a Fontan-type operation. Of the 336 patients who had a cavopulmonary shunt and were alive without having undergone a Fontan, the median age was 6.08 years (IQR 4.82–8.34 years): for some, the stage 2 may be definitive palliation.

Of the 212 patients who had a prepathway intervention, 123 continued to initial palliation, 56 skipped initial palliation and went straight to cavopulmonary shunt stage and 3 proceeded straight to the Fontan stage. There were 1140 patients who started their treatment pathway with initial palliation, 197 patients who started their treatment pathway with a cavopulmonary shunt stage and 8 patients had a Fontan-type palliation as their only pathway procedure.

Given that FUH is a diverse group, the initial palliation approaches varied, consisting of 537 (42.5%) systemic-to-pulmonary arterial shunts, 327 (25.9%) pulmonary arterial bands, 258 (20.4%) Norwood or Damus operations, 125 (9.9%) coarctation or interrupted arch repairs (with or without pulmonary trunk banding) and 16 (1.3%) hybrid procedures.

### Description of mortality outcomes

The outcomes shown in figure 3 are death in hospital following procedure, death after discharge or care ongoing/lost to follow-up. Heart transplants are represented with a heart symbol.

Table 2 shows patient characteristics and hospital stay within the tertiary centre for each stage. In-hospital mortality following prepathway intervention was 7.5% (95% CI 4.7% to 11.9%), initial palliation was 9.1% (95% CI 7.6% to 10.8%), cavopulmonary shunt stage was 2.9% (95% CI 2.1% to 4.0%) and Fontan stage was 1.5% (95% CI 0.8% to 2.6%). In the small group (15 patients) who had heart transplant, the in-hospital mortality was 26.7% (95% CI 10.9% to 52.0%).

### Procedures outside the staged treatment pathway

#### Prepathway procedures

A total of 212 patients (13.6% of the whole cohort) had one or more prepathway procedures, the most common being a balloon atrial septostomy (95). No clinical risk factors were linked to occurrence of a prepathway procedure.

#### Additional cardiac procedures

The rate of additional surgical procedures was 5 per 100 patient-years, the rate of interventional catheterisations was 7 per 100 patient-years and the highest rate was after initial palliation (online supplemental appendix 3, table A4). Of the 1263 patients with FUH who had initial palliation, 34.0% had at least one additional cardiac procedure (most common: additional systemic-to-pulmonary arterial shunt procedure) (online supplemental appendix 3, table A5). Of the 1202 patients with FUH who had a cavopulmonary shunt stage procedure, 29.5% had at least one subsequent additional cardiac procedure (most common: systemic-to-pulmonary collateral artery (major aortopulmonary collateral artery-related catheter). Of the 814 patients who had a Fontan-type procedure, 27.3% had at least one subsequent additional cardiac procedure (most common: closure of a Fontan fenestration).

### Analysis of risk factors for primary and secondary outcomes

#### Survival

**Figure 4:**
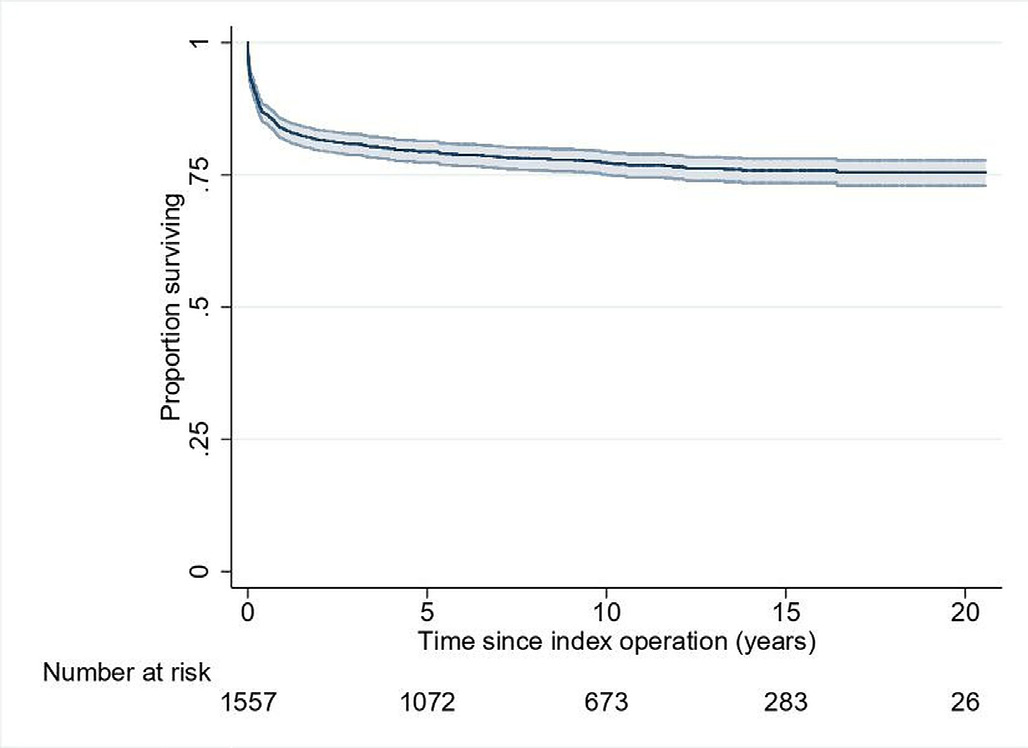
Kaplan-Meier survival curve for the whole cohort of patients with functionally univentricular heart, excluding hypoplastic left heart syndrome, with 95% CIs.

**Figure 5:**
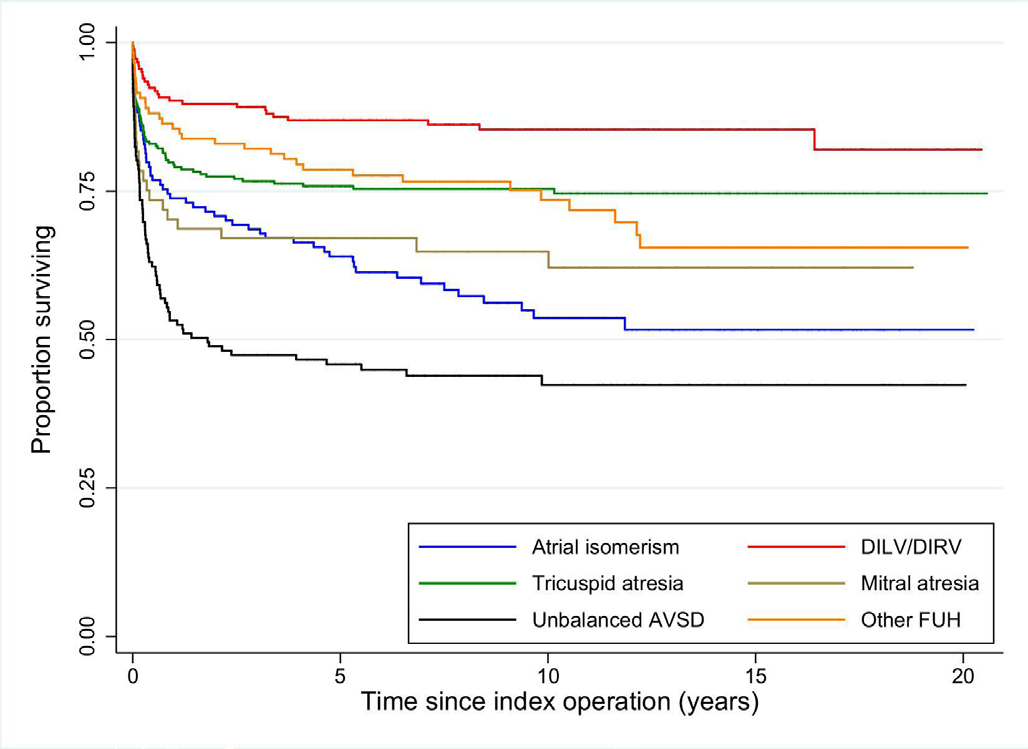
Risk-adjusted Kaplan-Meier survival curves for each functionally univentricular heart (FUH) subtype. AVSD, atrioventricular septal defect; DILV, double inlet left ventricle; DIRV, double inlet right ventricle.

The Kaplan-Meier survival curve for FUH (figure 4) shows the 1, 5 and 10-year survival rates after index operation were 83.6% (95% CI 81.7% to 85.4%), 79.4% (95% CI 77.3% to 81.4%) and 77.2% (95% CI 75.0% to 79.2%), respectively. The Kaplan-Meier survival curves for each FUH subtype are shown in figure 5.

The only patient factor that violated the proportional hazards assumption was the TA clinical subgroup. Thus, an extended multivariable Cox model with a time-dependent variable (ie, interaction of TA with follow-up time) was applied. The multivariable Cox model (table 1) showed that patients with DILV or DIRV were at lower risk of death (p<0.01) and patients with atrial isomerism (p<0.05) or unbalanced AVSD (p<0.001) were at higher risk of death than the middle reference group (other FUH including DORV). Patients with increased severity of illness (p<0.001), low weight (p<0.05) or an acquired comorbidity at index procedure (p<0.001) were at higher risk of death compared with the reference groups (none). Lastly, the risk of death for patients with TA (vs other FUH) decreased over time (p<0.01).

The sensitivity analyses did not change the statistical significance of the risk factors.

#### Additional cardiac procedures

As we show in table 3, patients with DILV or DIRV had a lower risk (p<0.01) and patients with antenatal diagnosis (p<0.01), congenital comorbidities (p<0.01) or acquired comorbidity at index procedure (p<0.001) had a higher risk of any additional cardiovascular procedures compared with reference (none). Increased severity of illness at index procedure added to the risk of additional cardiac procedures for unbalanced AVSD (p<0.05). Considering surgeries and interventional catheters separately: the risk factors for additional surgical procedures were the same as for ‘any type’ of additional procedure, except patients with MA were lower risk (p<0.05). The only significant risk factors for additional interventional catheter procedures were congenital comorbidity (p<0.01), acquired comorbidity (p<0.05) and increased severity of illness at index procedure (p<0.05).

**Table 3:** Univariable and multivariable incidence rate ratios (IRR) with 95% CIs of ‘any additional cardiac procedures’, ‘additional surgical procedures’ and ‘additional catheter procedures’

Risk factor	Any type of additional cardiac procedures		Additional surgical procedures		Additional catheter procedures	
	Univariable IRR (95% CI)	Multivariable IRR (95% CI)	Univariable IRR (95% CI)	Multivariable IRR (95% CI)	Univariable IRR (95% CI)	Multivariable IRR (95% CI)
**Clinical subgroups (Ref: other FUH)**						
FUH with atrial isomerism	1.10 (0.77 to 1.56)	1.04 (0.72 to 1.51)	1.10 (0.66 to 1.86)	0.96 (0.55 to 1.66)	1.10 (0.77 to 1.57)	1.13 (0.76 to 1.66)
DILV or DIRV	0.74 (0.54 to 1.02)	0.64 (0.46 to 0.90)**	0.64 (0.40 to 1.03)	0.50 (0.30 to 0.82)**	0.86 (0.62 to 1.18)	0.83 (0.58 to 1.17)
Tricuspid atresia	0.87 (0.64 to 1.18)	0.73 (0.53 to 1.02)	0.89 (0.56 to 1.41)	0.61 (0.37 to 1.01)	0.86 (0.63 to 1.18)	0.85 (0.60 to 1.21)
Mitral atresia	0.77 (0.50 to 1.20)	0.84 (0.52 to 1.34)	0.51 (0.26 to 1.00)	0.47 (0.23 to 0.99)*	1.06 (0.68 to 1.64)	1.22 (0.76 to 1.96)
Unbalanced AVSD	2.17 (1.49 to 3.18)	1.43 (0.96 to 2.13)	3.20 (1.81 to 5.66)	1.45 (0.78 to 2.71)	1.43 (0.99 to 2.07)	1.35 (0.90 to 2.00)
**Gender (Ref: female)**						
Male	1.05 (0.86 to 1.27)	1.09 (0.90 to 1.32)	0.97 (0.73 to 1.29)	1.03 (0.77 to 1.38)	1.09 (0.90 to 1.32)	1.12 (0.92 to 1.36)
**Index procedure weight <2.5 kg (Ref: weight >2.5 kg)**						
Yes	2.65 (1.78 to 3.93)	0.75 (0.31 to 1.83)	2.57 (1.43 to 4.65)	0.65 (0.17 to 2.48)	1.93 (1.31 to 2.83)	0.86 (0.35 to 2.15)
**Ethnicity (Ref: non-white)**						
White	1.18 (0.96 to 1.45)	1.14 (0.91 to 1.41)	1.13 (0.83 to 1.53)	1.17 (0.83 to 1.63)	1.15 (0.93 to 1.42)	1.10 (0.88 to 1.38)
**Antenatal diagnosis (Ref: none)**						
Yes	1.64 (1.30 to 2.06)	1.43 (1.12 to 1.82)**	1.92 (1.35 to 2.72)	1.67 (1.14 to 2.45)*	1.40 (1.11 to 1.77)	1.24 (0.96 to 1.59)
**Congenital non-cardiac comorbidity (Ref: none)**						
Yes	1.88 (1.48 to 2.40)	1.54 (1.20 to 1.97)**	1.95 (1.37 to 2.78)	1.57 (1.09 to 2.31)*	1.72 (1.35 to 2.19)	1.51 (1.17 to 1.94)**
**Prematurity (Ref: full term)**						
Yes	2.15 (1.48 to 3.13)	1.33 (0.87 to 2.04)	2.02 (1.15 to 3.54)	1.20 (0.60 to 2.41)	1.97 (1.37 to 2.83)	1.42 (0.94 to 2.13)
**Index procedure acquired comorbidity (Ref: none)**						
Yes	5.00 (2.94 to 8.49)	3.78 (2.17 to 6.59)***	6.84 (3.18 to 14.70)	6.83 (3.02 to 15.46)***	2.70 (1.59 to 4.59)	1.77 (1.02 to 3.08)*
**Index procedure increased severity of illness (Ref: none)**						
Yes	4.19 (2.95 to 5.94)	2.42 (0.97 to 6.03)	4.93 (2.91 to 8.38)	2.21 (0.53 to 9.14)	3.11 (2.23 to 4.33)	2.53 (1.06 to 6.07)*
**Index of Multiple Deprivation (Ref: most deprived)**						
Second most deprived	0.90 (0.70 to 1.17)	0.88 (0.69 to 1.13)	0.95 (0.65 to 1.40)	0.92 (0.63 to 1.35)	0.94 (0.72 to 1.22)	0.91 (0.70 to 1.17)
Mid-deprived	1.15 (0.86 to 1.53)	0.90 (0.68 to 1.19)	1.08 (0.71 to 1.65)	0.96 (0.62 to 1.48)	1.19 (0.89 to 1.58)	0.95 (0.71 to 1.27)
Second least deprived	0.83 (0.60 to 1.14)	0.74 (0.54 to 1.03)	0.79 (0.49 to 1.27)	0.63 (0.38 to 1.03)	0.89 (0.64 to 1.23)	0.91 (0.66 to 1.27)
Least deprived	0.85 (0.61 to 1.19)	0.95 (0.68 to 1.32)	0.79 (0.49 to 1.30)	1.03 (0.62 to 1.71)	0.94 (0.67 to 1.31)	0.98 (0.70 to 1.37)
Clinical subgroups (Ref: other FUH) * Index procedure weight <2.5 kg (Ref: weight >2.5 kg)						
FUH with atrial isomerism (interaction) Yes		0.72 (0.17 to 3.04)		0.42 (0.05 to 3.73)		0.97 (0.22 to 4.41)
DILV or DIRV (interaction) Yes		2.55 (0.73 to 8.94)		1.75 (0.26 to 11.74)		2.31 (0.68 to 7.81)
Tricuspid atresia (interaction) Yes		1.51 (0.50 to 4.53)		2.05 (0.36 to 11.50)		1.39 (0.47 to 4.15)
Mitral atresia *(interaction) Yes		0.86 (0.17 to 4.37)		2.05 (0.20 to 21.06)		0.46 (0.07 to 3.11)
Unbalanced AVSD (interaction) Yes		2.53 (0.61 to 10.51)		2.59 (0.31 to 21.50)		3.07 (0.72 to 13.07)
**Clinical subgroups (Ref: other FUH) (interaction) Index procedure increased severity of illness (Ref: none)**						
FUH with atrial isomerism (interaction) Yes		0.68 (0.17 to 2.77)		1.17 (0.15 to 9.39)		0.47 (0.11 to 1.99)
DILV or DIRV (interaction) Yes		1.07 (0.34 to 3.37)		0.92 (0.16 to 5.35)		1.18 (0.40 to 3.50)
Tricuspid atresia (interaction) Yes		1.66 (0.57 to 4.82)		3.08 (0.60 to 15.91)		0.94 (0.37 to 2.62)
Mitral atresia (interaction) Yes		0.53 (0.11 to 2.59)		0.35 (0.02 to 6.30)		0.67 (0.15 to 3.06)
Unbalanced AVSD (interaction) Yes		4.39 (1.10 to 17.45)*		12.04 (1.57 to 92.51)*		2.13 (0.56 to 8.06)

*P<0.05; **p<0.01; ***p<0.001.

AVSD, atrioventricular septal defect; DILV, double inlet left ventricle; DIRV, double inlet right ventricle; FUH, functionally univentricular heart.

The sensitivity analyses did not change the statistical significance of the risk factors.

## Discussion

### Summary of findings

Among the 53 615 patients born after 2000, who had intervention for CHD in England and Wales, 3% had FUH. The FUH group is characterised by highly variable morphology, as reflected by the outcomes by subtype, for example, although the average actuarial survival at 10 years after index operation was 77.2%, the HR for double inlet hearts was 0.49, whereas the HR for unbalanced AVSD (excluding atrial isomerism) was 2.75. It is possible that the higher mortality rate in unbalanced AVSD may relate to the adverse impacts of atrioventricular valve dysfunction in FUH. We were surprised that congenital non-cardiac anomalies were not linked to mortality, and we speculate that this may reflect more severe comorbidities and may have led to termination of pregnancy or abstention from surgical treatment. As for HLHS,^12^ additional cardiovascular procedures were frequently required for children with FUH, affecting approximately one-third of the cohort, especially among those who presented in poor condition in the neonatal period, likely because of a greater need for temporising interventions.

### Findings in context

There are few population-based longitudinal studies with which to compare our data. The Australia and New Zealand Fontan Registry publishes detailed outcome information, although related to patients who have already reached Fontan stage.^6^ Although this registry published outcomes by several comparable diagnostic groups, unlike our data, they observed no statistically different outcomes by diagnostic subgroup.^22^ A recent meta-analysis reported long-term survival post-Fontan for heterotaxy syndrome to be 74% at 10 years, consistent with our data.^23^

The government census (2011) found <4% of children in England and Wales were of South Asian ethnicity,^24^ and although the proportion of FUH with South Asian ethnicity in our cohort is not corrected for birth rate, at 18.3%, it appears considerably higher. Given that 78.6% of patients in our study were antenatally diagnosed, most families will have been offered the option of termination of pregnancy. People with South Asian heritage are more likely to live in a deprived neighbourhood in the UK and pregnancies have a greater risk of being affected by congenital anomalies^25^ and specifically by complex CHD.^17^ One explanation is that South Asian patients of Pakistani heritage have high rates of consanguinity.^26^ A recent study found that couples with consanguinity and Pakistani heritage were less likely to consent to invasive fetal testing and to genetic follow-up, which might make them more vulnerable to the risk of a pregnancy affected by congenital anomalies.^27^ Therefore, the higher than expected proportion of children with South Asian heritage in our study could result from a combination of genetic predisposition and also parental views about pregnancy termination. Studies from the USA found lower survival rates with CHD in Black patients,^28^ and with poorer socioeconomic circumstances in children with HLHS.^29^ It is possible that NHS care, which is free at the point of access, may offer an advantage in the long-term management of this very complex CHD.

We note that patients with FUH were of low weight for age at all surgical stages. Previous research has linked low weight to poorer outcomes among children with FUH, improved with feeding interventions,^30^ hence this topic merits further exploration in an additional future study.

### Limitations

As with any registry-based study, the retrospective analysis of an observational data set holds inherent limitations and reflects the data quality. We took an inclusive approach, retaining all patients where the diagnosis was consistent with FUH, irrespective of reasonable variations in the timing or types of procedures, since this represented our best assessment of the true picture of events. As stated, only patients who underwent at least one procedure are in the source data.

## Conclusion

Our population-based analysis of this highly complex and heterogeneous condition, FUH, reveals that patients from more deprived backgrounds and South Asian ethnicity are over-represented, and although nearly 8 out of 10 patients survive to 10 years, this is with a high burden of additional unplanned interventions.

**Key messages**

**What is already known on this subject?**

- Functionally univentricular heart (FUH) conditions are extremely complex and managed with staged surgical palliation developed for these conditions (Fontan pathway).
- Population-based data reporting longer term outcomes of FUH reflecting current practice are scarce.

**What might this study add?**

- Interventional treatment pathways followed for FUH are complex and highly variable.
- Patients of South Asian ethnicity and those living in the most deprived quintile areas are over-represented among those with this very complex heart condition.
- The 10-year survival was 77.2%, and 34.0% of children who underwent initial palliation had an additional cardiac intervention over and above their planned staged treatment.

**How might this impact on clinical practice?**

- Our data on longer term outcomes can be used to inform families during decision-making for their child, for example, during fetal counselling.
- It is essential that long-term outcomes of conditions like FUH that require serial interventions are used for audit to provide a fuller picture and to inform quality assurance and improvement.

## Data Availability

Data are available subject to legal data sharing agreements with the data providers (NCHDA, NHS Digital) and data sharing requires ethical approval and CAG approval. Study data are held subject to a data sharing agreements with the data providers. Requests for data sharing would be subject to approvals by the National Cardiac Audit Programme, the Health Quality Improvement Partnership and NHS Digital.
