# Supplementary material for: Cohort study of intervened functionally univentricular heart in England and Wales (2000–2018)
Source: Heart. 2021 Oct 27;108(13):1046–54. doi: 10.1136/heartjnl-2021-319677 (PMC9209673; doi:10.1136/heartjnl-2021-319677)
Supplement: Supplementary data [file heartjnl-2021-319677supp001.pdf]

Appendix 1

Inclusion and Exclusion criteria

1a) Algorithm summary for creation of FUH dataset

Step 1: In order to separate them as a group and exclude them from the FUH dataset, we identified patients with classic HLHS, based on presence of a hypoplastic left ventricle, aortic or mitral atresia, or procedure codes consistent with Norwood or hybrid procedures for HLHS, as reported in our previous paper. (13)

Step 2: We identified patients with HLHS codes present in Step 1, and at least one of the codes in Table A1, which are all codes inconsistent with classic HLHS, such as double inlet ventricle, double outlet right ventricle, and unbalanced atrioventricular septal defect (AVSD). In accordance with the ISNPCHD definition, the majority of these patients had HLHS related malformations. (11)

Step 3: We identified patients without HLHS or HLHS related malformations, with otherwise clear FUH anatomy (Table A2), such as tricuspid atresia, double inlet ventricle, and AVSD with ventricular imbalance and FUH procedural pathway. (15)

Step 4: We identified and excluded patients with specific primary CHD diagnoses in whom the degree of ventricular hypoplasia varies such that they may have been misallocated to the FUH group, solely due to the presence of a hypoplastic ventricle code (Table A3), such as pulmonary atresia with intact ventricular septum.

Step 5: We excluded patients from Step 4 (or elsewhere) with any procedure codes indicating a definitive biventricular repair was undertaken since this is inconsistent with FUH (Appendix 2A).

Step 6: After undertaking steps 1) to 5), we reviewed the remaining patients in the main NCHDA dataset with procedures indicative of a SV circulation (Appendix 2B). This identified further patients with FUH with minor coding errors and: a) codes from Table A2; b) codes indicating the presence of SV circulation and heterotaxy syndrome (e.g. right or left atrial isomerism) (19, 20); or c) codes indicated an unbalanced AVSD with SV circulation.

1b) Algorithm in detail including codes:

Step 1: Identify HLHS patients

Patients with at least one of the following diagnosis codes:

- 01.01.09: HLHS
- 09.15.03: Aortic atresia
- 06.02.01: Mitral atresia
- 07.08.42: Ventricular imbalance: dominant right ventricle and HLV
- 07.07.00: Left ventricular hypoplasia

or procedure codes consistent with Stage 1 Norwood procedure for HLHS:

- 12.10.00: Norwood type procedure
- 12.09.03: Damus-Kaye-Stansel type procedure: pulmonary trunk to aorta end/side anastomosis and at least one of the following procedure codes:
  12.31.03: Modified R Blalock shunt
  12.31.04: Modified L Blalock shunt

12.31.06: Central systemic-PA interposition shunt
12.31.46: Modified Blalock shunt
12.31.30: Systemic-to-pulmonary arterial shunt procedure

or procedure codes consistent with Stage 1 or Stage 2 hybrid for HLHS:

- 12.10.04: Application of bilateral pulmonary arterial bands & transcatheter placement of stent in arterial duct
- 12.14.19: Application of right & left pulmonary arterial bands and 12.10.14: stent placement in arterial duct within 4 weeks
- 12.20.20: Hypoplastic left heart syndrome hybrid approach (transcatheter & surgery): stage 1
- 12.20.21: Hypoplastic left heart syndrome hybrid strategy (transcatheter & surgery)
- 12.20.22: Hypoplastic left heart syndrome hybrid approach (transcatheter & surgery) 'stage 2': aortopulmonary amalgamation + superior cavopulmonary anastomosis(es) + debanding of pulmonary arteries
- 12.20.23: Hypoplastic left heart syndrome hybrid approach (transcatheter & surgery) 'stage 2': aortopulmonary amalgamation + superior cavopulmonary anastomosis(es) + debanding of pulmonary arteries + arch repair

and no diagnostic codes indicative of HLHS related malformations from Table A1.

Step 2: Identify HLHS related malformation patients

Patients with at least one of the following diagnosis codes:

- 01.01.09: HLHS
- 09.15.03: Aortic atresia
- 06.02.01: Mitral atresia
- 07.08.42: Ventricular imbalance: dominant right ventricle and HLV
- 07.07.00: Left ventricular hypoplasia

and at least one of the diagnostic codes from Table A1.

Step 3: Identify FUH patients

- Removal of any HLHS patients identified in Step 1.
- Inclusion of HLHS related malformation patients identified in Step 2.
- Inclusion of patients with at least one of the diagnosis codes from Table A2.

Step 4: Identify and exclude patients misallocated to the FUH group

- Exclusion of patients with at least one of the FUH exclusion diagnostic codes from Table A3.

Step 5: Exclude patients with any procedure codes from Appendix 2A.

Step 6: Identify any remaining patients in the main NCHDA dataset with procedures indicative of a SV circulation (Appendix 2B).

Table A1. Diagnostic codes that were combined with at least one of: HLHS, aortic atresia, mitral atresia, ventricular imbalance: dominant right ventricle and hypoplastic left ventricle, or left ventricular hypoplasia. Presence of one of these codes led to the patient being excluded from the Classic HLHS Group.

03.01.05	Left isomerism ('polysplenia')
03.01.04	Right isomerism ('asplenia')
01.03.09	Atrioventricular and-or ventriculo-arterial connections abnormal
01.01.14	Double inlet atrioventricular connection (double inlet ventricle)
01.04.03	Double inlet right ventricle
01.04.04	Double inlet left ventricle
06.01.01	Tricuspid atresia
01.05.01	Discordant ventriculo-arterial connections (TGA)
01.01.02	Transposition of great arteries (TGA) (concordant atrioventricular & discordant ventriculo-arterial connections) & intact ventricular septum
01.01.03	Congenitally corrected transposition of great arteries (discordant atrioventricular & ventriculo-arterial connections)
01.01.04	Double outlet right ventricle
01.01.17	Double outlet right ventricle: Fallot type (subaortic or doubly committed ventricular septal defect & pulmonary stenosis)
01.01.40	Double outlet right ventricle: subaortic or doubly committed ventricular septal defect without pulmonary stenosis ('VSD type')
01.01.18	Double outlet right ventricle: transposition type (subpulmonary ventricular septal defect)
01.01.19	Double outlet right ventricle: with non-committed ventricular septal defect
01.01.24	Double outlet right ventricle: with intact ventricular septum
01.05.03	Double outlet left ventricle
09.01.01	Common arterial trunk (truncus arteriosus)
09.05.11	Pulmonary atresia
09.05.12	Pulmonary atresia: imperforate valve
01.01.07	Pulmonary atresia + intact ventricular septum
01.01.06	Pulmonary atresia + ventricular septal defect (including Fallot type)
01.01.25	Pulmonary atresia + ventricular septal defect + systemic-to-pulmonary collateral artery(ies) (MAPCA(s))
06.02.09	Straddling mitral valve
06.06.00	Atrioventricular septal defect (AVSD)

06.06.01	Atrioventricular septal defect: isolated atrial component (primum ASD) (partial AVSD)
06.06.08	Atrioventricular septal defect: isolated ventricular component
06.06.10	Atrioventricular septal defect: atrial & (restrictive) ventricular components + separate atrioventricular valve orifices ('intermediate')
06.06.09	Atrioventricular septal defect: atrial & ventricular components with common atrioventricular orifice (complete)
01.01.20	Atrioventricular septal defect and tetralogy of Fallot
06.07.26	Atrioventricular septal defect with ventricular imbalance
06.05.01	Atrioventricular septal defect atrioventricular valvar abnormality
06.05.06	Atrioventricular septal defect atrioventricular valvar regurgitation
07.08.41	Ventricular imbalance: dominant left ventricle + hypoplastic right ventricle
07.02.00	Right ventricular hypoplasia

Table A2. FUH diagnostic codes

01.01.14	Double inlet atrioventricular connection (double inlet ventricle)
01.01.22	Functionally univentricular heart
01.01.24	Double outlet right ventricle with intact ventricular septum
01.04.03	Double inlet right ventricle
01.04.04	Double inlet left ventricle
02.03.05	Solitary ventricle of indeterminate morphology
06.01.01	Tricuspid atresia
06.07.26	Atrioventricular septal defect with ventricular imbalance
07.08.41	Ventricular imbalance: dominant left ventricle + hypoplastic right ventricle
07.08.42	Ventricular imbalance: dominant right ventricle and HLV

Table A3. FUH exclusion diagnostic codes.

01.01.07	Pulmonary atresia + intact ventricular septum
01.01.25	Pulmonary atresia + ventricular septal defect + systemic-to-pulmonary collateral artery(ies) (MAPCA(s))
01.01.20	Atrioventricular septal defect and Tetralogy of Fallot
09.05.25	Absent pulmonary valve syndrome
09.01.01	Common arterial trunk (truncus arteriosus)

Appendix 1c)

Procedures indicative of a definitive biventricular repair

- 12.00.17: Scimitar syndrome (partially anomalous pulmonary venous connection) repair
- 12.00.29: Systemic venous pathway procedure (post Senning-Mustard)
- 12.01.07: Patent foramen ovale (PFO) closure with transluminal device
- 12.01.53: Patent foramen ovale (PFO) direct closure
- 12.01.98: Interatrial communication closure with transluminal device
- 12.04.01: Atrioventricular septal defect (AVSD): partial (primum ASD) repair
- 12.04.09: Atrioventricular septal defect (AVSD): partial with isolated ventricular component (VSD) repair
- 12.05.01: Atrioventricular septal defect (AVSD): complete (common valve orifice) repair
- 12.05.10: Atrioventricular septal defect (AVSD): 'intermediate' repair
- 12.06.35: Double chambered right ventricle repair
- 12.07.19: Left ventricular outflow tract obstruction relief by transcatheter coronary chemical ablation
- 12.07.38: Partial left ventriculectomy-volume reduction (Batista)
- 12.08.01: Ventricular septal defect (VSD) closure
- 12.08.02: Ventricular septal defect (VSD) closure by direct suture
- 12.08.03: Ventricular septal defect (VSD) closure using patch
- 12.08.07: Ventricular septal defect (VSD) closure with transluminal device
- 12.08.16: Closure of multiple ventricular septal defect (VSD)s
- 12.08.19: Open fenestration of ventricular septal defect (VSD) patch
- 12.08.20: Transluminal fenestration of ventricular septal defect (VSD) patch
- 12.08.28: Intraoperative ventricular septal defect (VSD) closure with transluminal device (hybrid approach)
- 12.11.00: Common arterial trunk (truncus) repair
- 12.12.01: Aortopulmonary window closure
- 12.13.21: Pulmonary valvar replacement (not conduit)
- 12.13.22: Pulmonary valvar replacement using homograft
- 12.13.51: Transluminal pulmonary valvar insertion with stent mounted valve
- 12.13.55: Pulmonary valve repair converted to pulmonary valvar replacement
- 12.13.81: Transluminal aortic valvar insertion with stent mounted valve
- 12.13.84: Transapical aortic valve implantation (hybrid approach)
- 12.13.85: Transluminal pulmonary valvar insertion with stent mounted valve including prestenting
- 12.13.86: Transluminal pulmonary valvar prestenting procedure in preparation for valve replacement
- 12.14.30: Pulmonary artery origin from ascending aorta (hemitruncus) repair
- 12.16.30: Ross procedure: aortic valve or root replacement with pulmonary autograft & pulmonary valvar replacement
- 12.16.62: Ross-Konno procedure
- 12.16.81: Aortic sinus of Valsalva distal fistula closure
- 12.16.85: Aortic sinus of Valsalva aneurysm repair
- 12.16.90: Aorto-left ventricular tunnel closure
- 12.17.32: Pulmonary arterial sling repair
- 12.17.99: Aortic root replacement of implanted pulmonary autograft & pulmonary valve re-replacement
- 12.23.00: Anomalous coronary artery (eg ALCAPA) repair

- 12.23.42: Transluminal chemical occlusion of coronary artery
- 12.23.80: Anomalous aortic origin of coronary artery repair
- 12.26.01: Tetralogy of Fallot repair
- 12.26.13: Tetralogy of Fallot repair with transannular patch
- 12.26.20: Tetralogy of Fallot repair without transannular patch
- 12.26.21: Absent pulmonary valve syndrome (Fallot type) repair
- 12.27.01: Double outlet right ventricle with subaortic or doubly committed ventricular septal defect (VSD) & pulmonary stenosis (Fallot-type) repair
- 12.27.02: Double outlet right ventricle repair with intraventricular tunnel
- 12.27.45: REV procedure: intraventricular left ventricle to aorta tunnel with infundibular septum resection & direct right ventricle to pulmonary trunk anastomosis
- 12.27.50: Double outlet left ventricle repair
- 12.27.78: Aortic root translocation to over left ventricle (including Nikaidoh)
- 12.28.01: Pulmonary atresia & ventricular septal defect (VSD) (including Fallot-type) repair
- 12.28.11: Pulmonary atresia, ventricular septal defect (VSD) & systemic-to-pulmonary collateral artery(ies) (MAPCA(s)) repair
- 12.29.01: Senning procedure (atrial inversion)
- 12.29.02: Mustard procedure (atrial inversion)
- 12.29.20: Double outlet right ventricle repair
- 12.29.21: Arterial switch procedure
- 12.29.25: Arterial switch & atrial inversion procedures ('double switch')
- 12.29.26: Atrial inversion and Rastelli procedures
- 12.29.52: Pulmonary venous pathway procedure (post Senning-Mustard)
- 12.29.79: Atrial inversion procedure (Mustard or Senning) revision
- 12.37.60: Lung(s) transplant
- 12.38.25: Transluminal left atrial appendage occlusion with device

Appendix 1d)

Procedures indicative of a SV circulation

- 12.02.70: Tricuspid valvar closure
- 12.02.77: Ebstein malformation of tricuspid valve repair: tricuspid valve closure & shunt (Starnes)
- 12.10.00: Norwood type procedure
- 12.16.61: Aortic valve closure-oversewing
- 12.20.20: Hypoplastic left heart syndrome hybrid approach (transcatheter & surgery): stage 1
- 12.20.21: Hypoplastic left heart syndrome hybrid strategy (transcatheter & surgery)
- 12.20.22: Hypoplastic left heart syndrome hybrid approach (transcatheter & surgery) 'stage 2': aortopulmonary amalgamation + superior cavopulmonary anastomosis(es) + debanding of pulmonary arteries
- 12.20.23: Hypoplastic left heart syndrome hybrid approach (transcatheter & surgery) 'stage 2': aortopulmonary amalgamation + superior cavopulmonary anastomosis(es) + debanding of pulmonary arteries + arch repair
- 12.30.01: Fontan type procedure
- 12.30.05: Total cavopulmonary connection (TCPC) using extracardiac inferior caval vein (IVC)-pulmonary artery conduit with fenestration
- 12.30.06: Total cavopulmonary connection (TCPC) with fenestrated lateral atrial tunnel
- 12.30.13: Fontan procedure with atrioventricular connection
- 12.30.27: Fenestration of Fontan type connection

- 12.30.28: Fontan-type connection without fenestration
- 12.30.32: Fontan procedure with direct atriopulmonary anastomosis
- 12.30.34: Conversion of Fontan repair to total cavopulmonary connection
- 12.30.37: Fontan type procedure revision or conversion
- 12.30.50: Total cavopulmonary connection (TCPC)
- 12.30.51: Total cavopulmonary connection (TCPC) with lateral atrial tunnel
- 12.30.54: Total cavopulmonary connection (TCPC) using extracardiac inferior caval vein (IVC)-pulmonary artery conduit
- 12.30.56: Takedown of total cavopulmonary connection (TCPC)
- 12.30.60: Completion of total cavopulmonary connection (TCPC) using transcatheter inferior to superior caval vein covered stent
- 12.31.15: Hemi-Fontan procedure
- 12.46.00: Atrioventricular valvar procedure in double inlet ventricle

Appendix 2

Categorizations of initial palliation procedures

A: Norwood/Damus

- 12.10.00: Norwood type procedure
- 12.09.03: Damus-Kaye-Stansel type procedure: pulmonary trunk to aorta end/side anastomosis

B: Coarctation/interrupted arch repair

- 12.18.00: Coarctation-hypoplasia of aorta repair
- 12.21.00: Interrupted aortic arch repair
- 12.18.10: Aortic coarctation-hypoplasia repair by resection & extended end to end anastomosis
- 12.18.01: Aortic coarctation-hypoplasia repair by resection & end to end anastomosis
- 12.18.02: Aortic coarctation-hypoplasia repair by patch aortoplasty
- 12.18.03: Aortic coarctation-hypoplasia repair by subclavian flap aortoplasty
- 12.18.15: Aortic coarctation-hypoplasia repair by resection & insertion of tube graft
- 12.18.30: Aortic arch repair
- 12.18.04: Balloon dilation of native aortic coarctation-hypoplasia
- 12.18.27: Aortic coarctation transluminal obstruction relief

C: Hybrid: PDA stent and Bilateral PA Bands

- 12.10.04: Application of bilateral pulmonary arterial bands & transcatheter placement of stent in arterial duct
- 12.14.19: Application of right & left pulmonary arterial bands and 121014: Stent placement in arterial duct within 4 weeks
- 12.20.20: Hypoplastic left heart syndrome hybrid approach (transcatheter & surgery): stage 1
- 12.20.21: Hypoplastic left heart syndrome hybrid strategy (transcatheter & surgery)

D: Securing pulmonary blood flow, e.g. systemic-to-pulmonary arterial shunt

- 12.31.03: Modified R Blalock interposition shunt
- 12.31.04: Modified L Blalock interposition shunt
- 12.31.06: Central systemic-PA interposition shunt
- 12.31.46: Modified Blalock interposition shunt
- 12.31.30: Systemic-to-pulmonary arterial shunt procedure
- 12.06.43: Right ventricle to pulmonary artery valveless conduit construction (Japanese modification: 'Sano') as part of Norwood procedure
- 12.13.02: Pulmonary valvotomy: open
- 12.08.21: Subpulmonary obstruction relief
- 12.10.14: Stent placement in arterial duct (PDA)
- 12.06.18: Stent placement in right ventricular outflow tract
- 12.06.41: Right ventricular outflow tract obstruction relief
- 12.36.01: Right ventricle to pulmonary arterial tree conduit construction
- 12.31.05: Waterston (ascending aorta-right pulmonary artery) anastomosis
- 12.13.05: Balloon dilation of pulmonary valve
- 12.13.09: Pulmonary valvar transluminal perforation & dilation

E: Protecting pulmonary vascular bed from excessive flow, e.g. Pulmonary trunk Band

- 12.14.02: Pulmonary trunk band (PA band)

Appendix 3

Supplementary Results

Table A4. Rate of off-pathway procedures per 100 patient-years at the different stages of the FUH treatment.

	**Total follow-up from primary procedure (per 100 patient-years)**	**Post initial palliation (per 100 patient-years)**	**Post cavopulmonary shunt stage (per 100 patient-years)**	**Post Fontan stage (per 100 patient-years)**
Surgical procedures	5	22	4	1
Interventional catheter procedures	7	15	7	4
Total	12	37	11	5

Table A5. Frequency of additional cardiac interventions of different types by the stages of FUH treatment. Procedures may arise more than once in the same patient.

	Intervention type	Pre-pathway Number (%)	Post initial palliation Number (%)	Post cavopulmonary shunt stage Number (%)	Post Fontan stage Number (%)	Post Heart Transplant Number (%)
Surgical procedures	Atrial septectomy/enlargement	44 (18.0)	19 (3.9)	2 (0.5)	2 (0.7)	0
	Damus-Kaye-Stansel type procedure	*0*	*18 (3.7)*	*2 (0.5)*	*1 (0.3)*	*0*
	Systemic-to-pulmonary arterial shunt	*0*	*98 (20.2)*	*8 (1.8))*	*0*	*0*
	Pulmonary trunk band (PA band)	0	54 (11.1)	8 (1.8)	0	0
	Repair of total anomalous pulmonary venous connection	14 (5.7)	5 (1.0)	2 (0.5)	0	0
	Patent ductus arteriosus ligation	3 (1.2)	5 (1.0)	0	0	0
	Bilateral pulmonary arterial bands	9 (3.7)	0	0	0	0
		0	14 (2.9)	0	0	0

	Norwood redo procedure					
	Bidirectional cavopulmonary shunt (Glenn) redo	0	0	15 (3.4)	0	0
	Takedown of Bidirectional cavopulmonary shunt	0	0	9 (2.0)	0	0
	Atrioventricular valve replacement or repair	1 (0.4)	3 (0.6)	10 (2.3)	1 (0.3)	0
	Pulmonary arterioplasty/reconstruction	0	9 (1.9)	10 (2.3)	4 (1.4)	0
	Fontan takedown or redo	0	0	0	17 (5.8)	0
	Procedure involving systemic vein	0	1 (0.2)	3 (0.7)	2 (0.7)	0
	Removal or adjustment of pulmonary arterial band(s)	0	14 (2.9)	0	0	0
	Cardiac conduit procedure	0	23 (4.7)	0	1 (0.3)	0
	Creation of Fontan fenestration	0	0	0	3 (1.0)	0
	Complex or not fully described procedures	7 (2.9)	7 (1.4)	15 (3.9)	0	0
	Pacemaker	6 (2.5)	21 (4.3)	20 (4.5)	33 (11.3)	0
Cat het er	Balloon atrial septostomy	134 (54.9)	38 (7.8)	0	0	

						0
	Recoarctation angioplasty	0	41 (8.5)	13 (2.9)	0	0
	Balloon dilation of pulmonary artery	0	26 (5.4)	60 (13.5)	17 (5.8)	0
	Pulmonary artery stenting	2 (0.8)	17 (3.5)	50 (11.3)	33 (11.3)	0
	Patent ductus arteriosus stent	2 (0.8)	10 (2.1)	0	0	0
	Systemic-to-pulmonary collateral artery (MAPCA) related catheter procedure	2 (0.8)	10 (2.1)	70 (15.8)	23 (7.9)	0
	Radiofrequency ablation for tachyarrhythmia	1 (0.4)	3 (0.6)	11 (2.5)	0	0
	Veno-venous collateral occlusion with device	0	0	24 (5.4)	0	0
	Shunt or conduit interventions	0	17 (3.5)	3 (0.7)	8 (2.7)	0
	Closure of Fontan fenestration	0	0	0	70 (24.0)	0
	Creation of a Fontan fenestration	0	0	0	23 (7.9)	0
	Procedure involving systemic vein	0	2 (0.4)	40 (9.0)	15 (5.1)	0

	Closure of systemic to pulmonary shunt	0	0	8 (1.8)	2 (0.7)	0
	Complex or not fully described procedures	19 (7.8)	30 (6.2)	60 (13.5)	37 (12.7)	5 (100.0)
	**Total**	**244**	**485**	**443**	**292**	**5**

Table A6 Stage one procedure types distribution based on the FUH diagnostic subgroups

Type of stage one palliation	Single ventricle with atrial isomerism	DILV/DIRV	TA	MA	Unbalanced AVSD	Other FUH	Total
Surgery includes Norwood type repair, a hybrid type procedure or a coarctation repair (Groups A+B+C)	29 (13.3)	134 (39.1)	62 (13.8)	32 (30.8)	115 (48.7)	58 (28.2)	430 (27.6)
Provision of secure pulmonary blood flow via shunt or conduit (Group D)	113 (51.8)	79 (23.0)	229 (50.9)	36 (34.6)	46 (19.5)	74 (35.9)	577 (37.1)
Protection of pulmonary blood flow (banding Group E)	35 (16.1)	70 (20.4)	80 (17.8)	22 (21.2)	58 (24.6)	46 (22.3)	311 (20.0)
None of the above	41 (18.8)	60 (17.5)	79 (17.6)	14 (13.5)	17 (7.2)	28 (13.6)	239 (15.4)
Total	218 (100.0)	343 (100.0)	450 (100.)	104 (100.0)	236 (100.0)	206 (100.0)	1,557 (100.0)
